# Microbial Proteins: A Green Approach Towards Zero Hunger

**DOI:** 10.3390/foods14152636

**Published:** 2025-07-28

**Authors:** Ayesha Muazzam, Abdul Samad, AMM Nurul Alam, Young-Hwa Hwang, Seon-Tea Joo

**Affiliations:** 1 Division of Applied Life Science (BK21 Four), Gyeongsang National University, Jinju 52828, Republic of Korea; ashu2nice@gmail.com (A.M.); buzdarabdulsamad@gmail.com (A.S.); alam6059@yahoo.com (A.N.A.); 2 Institute of Agriculture & Life Science, Gyeongsang National University, Jinju 52828, Republic of Korea; philoria@gnu.ac.kr

**Keywords:** microbial proteins, meat alternatives, food security, sustainable protein sources, UN sustainable development goals

## Abstract

The global population is increasing rapidly and, according to the United Nations (UN), it is expected to reach 9.8 billion by 2050. The demand for food is also increasing with a growing population. Food shortages, land scarcity, resource depletion, and climate change are significant issues raised due to an increasing population. Meat is a vital source of high-quality protein in the human diet, and addressing the sustainability of meat production is essential to ensuring long-term food security. To cover the meat demand of a growing population, meat scientists are working on several meat alternatives. Bacteria, fungi, yeast, and algae have been identified as sources of microbial proteins that are both effective and sustainable, making them suitable for use in the development of meat analogs. Unlike livestock farming, microbial proteins produce less environmental pollution, need less space and water, and contain all the necessary dietary components. This review examines the status and future of microbial proteins in regard to consolidating and stabilizing the global food system. This review explores the production methods, nutritional benefits, environmental impact, regulatory landscape, and consumer perception of microbial protein-based meat analogs. Additionally, this review highlights the importance of microbial proteins by elaborating on the connection between microbial protein-based meat analogs and multiple UN Sustainable Development Goals.

## 1. Introduction

The world population is increasing rapidly and, according to the United Nations, it is projected to reach approximately 9.8 billion by 2050 [1,2]. As the world’s population rises, the demand for food resources increases, along with other challenges, such as food shortages, less available land, low resources, and climate change [3]. This review aims to explore the current state of microbial protein production, its environmental and nutritional benefits, and the technological advancements necessary to overcome the scalability challenges. By examining these critical aspects, this review seeks to offer insights that will aid policymakers, researchers, and industry stakeholders in navigating the future of microbial proteins as a key element in global food security. Sustainable and innovative, environmentally friendly ideas are needed to handle these problems. Food security seriously needs attention [4]. Animal products like meat are very important; the main challenge in regard to food security now is to ensure that enough of these products are supplied. The demand for meat is increasing with the increasing population [5]. Meat alternatives can be a promising solution for the growing demand [6]. Plant-based meat [7,8], cultured meat [9,10], hybrid cultured meat [11], restructured meat [12], and microbial protein-based meat [13] are considered to be potential meat alternatives.

Bacteria, fungi, yeast, and algae-based approaches to producing microbial proteins are considered to be a sustainable method to develop meat analogs [14]. Companies that produce microbial protein need less space and water, and provide a nutritious diet to consumers [15]. These microbial proteins can also match the desired nutritional profile, using restructuring production technology to form meat analogs [12]. Microbial proteins as a food are a good choice for the circular economy, which values resource efficiency and reductions in waste. Microbes can convert food waste into proteins [16], and that protein can be utilized to make microbial protein-based meat alternatives. Traditional meat production, reliant on extensive land and water resources, is becoming increasingly unsustainable, instigating the search for innovative alternatives that meet both the nutritional needs and environmental goals of the future. While microbial proteins present an innovative solution to these challenges, their integration into global food systems faces several barriers, including high production costs, limited consumer awareness, and regulatory hurdles. Despite the promising environmental and nutritional benefits, a thorough understanding of microbial protein production, its scaling potential, and consumer perceptions is still needed.

The consumption of meat alternatives (e.g., plant-based meat) is growing rapidly [17]. Still, there are several challenges in regard to producing microbial protein-based meat analogs, e.g., cost, taste, and the final product’s appearance. It is essential to address these challenges to enhance the microbial protein-based meat analogs market. The use of restructuring technology can be a sustainable method to make restructured hybrid meat, which involves improving the texture, taste, and decreasing the cost [12]. This hybrid meat may contain plant-based proteins, animal-based proteins, or microbial proteins. Furthermore, this technology can be used to specify the nutritional profile of hybrid meat [12].

This review highlights all the significant aspects of microbial proteins and addresses the impact of microbial proteins on global food security and health, as well as the environmental benefits of microbial protein-based meat analogs. This review points out the importance of microbial proteins in achieving several UN Sustainable Development Goals, including SDG 2, SDG 12, SDG 13, SDG 15, and SDG 9. This review is conducted to share insights on microbial proteins with policymakers, researchers, and industry stakeholders, to shed light on the possibilities and difficulties of using microbial proteins in the global food system.

## 2. Microbial Proteins and the United Nations Sustainable Development Goals

**Figure 1:**
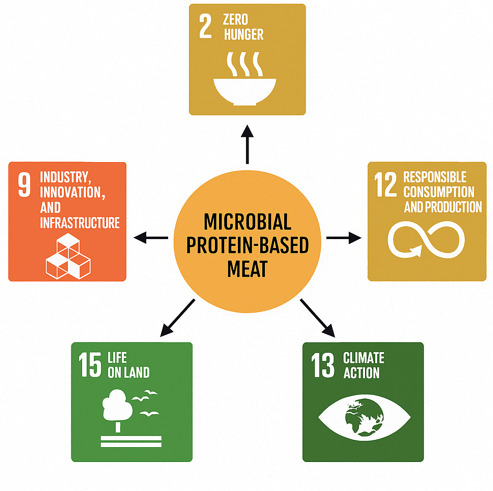
Link between microbial proteins and different SDGs set by the United Nations.

There are 17 Sustainable Development Goals and 169 agendas set by the UN that need proper attention. Microbial protein-based alternatives can be crucial in achieving five UN Sustainable Development Goals related to hunger, responsible consumption and production, climate action, life on land, and industry innovation. Table 1 and Figure 1 briefly elaborate on the relationship of these goals with microbial protein-based meat analogs.

## 3. Common Sources of Microbial Proteins

Microbial proteins, called single-cell proteins (SCPs), are biomass, protein extracts, or protein-rich products derived from microorganisms, such as bacteria, fungi, yeasts, and algae [23]. These microorganisms have a high protein content and can be cultivated using fermentation technologies, often on sustainable and low-cost substrates [24]. Microbial proteins offer an efficient and scalable solution for alternative protein production, addressing food security and environmental challenges [25]. Furthermore, microbes are rich in protein, while Table 2 presents a comparison of the protein ratios among various microbial protein sources. Microbial proteins can be derived from various microorganisms, including bacteria, fungi, yeasts, and algae. Each group has distinct characteristics that make it suitable for food applications. Among the different microbial proteins, bacteria have the highest protein content, ranging from 50 to 80%, making them ideal for high-yield production [26]. Algae come second with 50–70% protein, offering additional benefits like omega-3 fatty acids and antioxidants [27]. Yeasts provide moderate protein content (45–65%) and are also rich in B-vitamins [28], while fungi, with 40–60% protein, are valued for their meat-like texture and high fiber content, especially beta-glucans. All these microbial sources are complete proteins, containing all the essential amino acids, which makes them nutritionally valuable compared to other protein sources [29].

## 4. Sensory Properties of Microbial Proteins

**Texture:** Microbial proteins, particularly the mycoprotein of *Fusarium venenatum*, have a fibrous texture, which can be identical to meat [30]. It has been demonstrated that these proteins can be made to be meat-like in texture under the influence of extrusion or restructuring methods, which make them suitable for use as meat analogs [30]. Other proteins are produced by microbes, such as yeast and bacteria, including *Saccharomyces cerevisiae* and *Methylophilus methylotrophus*, which can also be used as an alternative to meat after processing. Humpenöder et al. [31] stated that mycoprotein offers a similar texture and protein quality to beef, with up to 96% lower CO_2_ emissions. Furthermore Elhalis et al. [32] stated that fermentation can be used to improve the sensory quality of plant-based meat analogs using fungi like *Fusarium venenatum* and bacteria like *Lactobacillus plantarum*.

**Color:** Microbial proteins occur in natural colors, dependent on their origin [33]. Protein sources like *Fusarium venenatum* are frequently beige [34], and algae sources like *Spirulina* provide a natural green color [35]. Beet juice and other natural colorants are occasionally added when making meat analogs using microbial proteins. Yeast and fungi, along with red yeast, are important in terms of the color and visual appeal of meat alternatives. Yeast, such as *Saccharomyces cerevisiae*, typically has a light beige or yellowish color, which may not naturally resemble the rich tones of meat. However, this can be adjusted with natural colorants like beetroot powder or caramel coloring to achieve a more meat-like appearance. On the other hand, fungi, such as *Fusarium venenatum* (used in Quorn) [36], generally have a pale off-white or ivory color, and, like yeast, they often require color modification to replicate the red, brown, or pink hues of meat, especially when used in products like vegan burgers [28]. Wu et al. [37] stated that red yeast, from *Monascus* species, naturally provides a red or reddish color, mimicking the pinkish hues of meat and is particularly beneficial in creating realistic meat analogs. The combination of yeast and fungi with natural colorants can help produce a realistic, appetizing appearance of meat substitutes, while color adjustments also ensure consumer appeal and acceptance.

**Flavor:** Yeast and other fungal microbial proteins contain high amounts of glutamic acid, which is responsible for generating an umami taste [38], and they can offer a taste similar to meat. For example, Quorn*(TM), a popular product manufactured using mycoprotein, has over the years enhanced its taste in order to enhance its acceptability in the meat alternatives market. Borthakur et al. [39] stated that *Fusarium venenatum* produces esters, aldehydes, ketones, and sulfur compounds that mimic savory, umami, and meaty notes; furthermore, Sharma et al. [40] have also reviewed the effect of microbes on flavor.

## 5. Nutritional and Functional Properties of Microbial Proteins

Microbial proteins derived from bacteria, fungi, yeasts, and algae offer a rich source of essential nutrients and functional properties that make them viable alternatives to conventional animal and plant proteins. Their high protein content, balanced amino acid profile, good digestibility, and additional bioactive compounds contribute to their potential role in addressing global nutritional needs. Microbial protein usually contains 40–80% protein, which is much higher than soy (30–40%) and beef (30–40%). Microbial protein is a complete protein rich in nine essential amino acids and is particularly high in lysine and threonine. Notably, microbial protein also contains a large amount of glutamic acid, which gives it a unique flavor, making it suitable for extracting umami peptides, such as those identified from spirulina protein (including the sequence of the umami peptide). Furthermore, the nutritional profile of microbial protein-based meat is briefly explained below and in Table 3.

## 6. Technologies for Microbial Protein Production

Microbial protein production relies on biotechnological advancements that optimize growth conditions, maximize yield, and ensure sustainability [50]. Various fermentation-based techniques and substrate utilization strategies are employed to enhance the efficiency and scalability of microbial protein production [51].

### 6.1. Fermentation-Based Approaches

Fermentation is the primary method for producing microbial proteins [52]. It involves cultivating microorganisms in a controlled environment to maximize biomass production [43]. Several types of fermentation, explained below, are used to produce microbial protein.

Through fermentation, these substrates can yield a range of high-value products, such as single-cell proteins (SCPs), enzymes, organic acids (e.g., lactic acid, citric acid, succinic acid), bioethanol, bioplastics (e.g., PHAs), bioactive peptides, and natural colorants. For example, orange peels can be used to produce pectinase and citric acid, while wheat bran can be used to support the production of xylanase, an enzyme used in food processing and animal feed.

These fermentation products have significant economic value, finding applications in the food and beverage industry, animal feed, pharmaceuticals, cosmetics, biofuel, and biodegradable packaging sectors. For instance, Danone and Unilever have explored microbial protein production from food processing waste, while companies like Cargill and ADM are using corn stover and molasses in fermentation to produce organic acids and amino acids.

Moreover, utilizing waste materials not only reduces raw material costs, but also contributes to a circular bioeconomy by converting low-value or polluting residues into valuable, marketable products. This waste valorization approach supports both environmental sustainability and economic efficiency, providing a dual benefit of reducing waste disposal issues, while generating profit [53].

#### 6.1.1. Submerged Fermentation (SmF)

Submerged Fermentation is the most widely used technique for the production of microbial protein. Microorganisms grow in liquid nutrient media, where the temperature, pH, aeration, and agitation are controlled to optimize the biomass yield [54]. This type of fermentation is mainly used for *Fusarium venenatum* (Quorn), *Saccharomyces cerevisiae*, and *Methylobacterium methylotrophus*.

#### 6.1.2. Solid-State Fermentation (SSF)

Solid-state fermentation (SSF) involves the growth of microorganisms on solid substrates in the absence of free-flowing water [55]. This technique is highly sustainable as it utilizes agro-industrial waste (e.g., wheat bran, corn stover). Solid-state fermentation is frequently employed in the cultivation of filamentous fungi like *Aspergillus oryzae* and *Rhizopus oligosporus*.

#### 6.1.3. Gas Fermentation

Certain bacteria, such as *Cupriavidus necator*, can convert gases like CO_2_, CH_4_, or H_2_ into proteins. Gas fermentation is a promising approach for sustainable, large-scale protein production, with minimal environmental impact [56]. For example, Calysta’s FeedKind uses methane as a feedstock to produce microbial protein for animal feed.

### 6.2. Single-Cell Protein (SCP) Production

**Figure 2:**
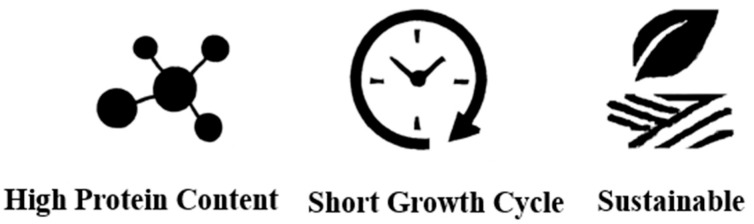
Advantages of SCP production.

SCP production refers to microbial biomass used directly as a protein source for food or feed [57]. The efficiency of SCP production depends on the microorganism used and the fermentation conditions. Furthermore, the advantages of SCP production are graphically explained in Figure 2, and the commonly used microbes for SCP production are presented in Table 4. In addition to this, a comparison of the different fermentation types that are being used in the production of microbial protein-based meat analogs is presented in Table 5.

### 6.3. Bioprocessing Using Waste Streams and Renewable Substrates

Using alternative feedstocks, such as agricultural residues, industrial byproducts, and renewable substrates, enhances the sustainability of microbial protein production [60].

#### 6.3.1. Agricultural and Food Waste

Agro-industrial waste materials, such as wheat straw, sugarcane bagasse, fruit peels, corn cobs, potato peels, spent coffee grounds, and brewery spent grains are rich in carbohydrates, proteins, fibers, and micronutrients, making them promising substrates for microbial fermentation [61]. These materials can be hydrolyzed into fermentable sugars, amino acids, and other nutrients that support the growth of various microorganisms, including bacteria (*Bacillus subtilis, Lactobacillus* spp.), fungi (*Aspergillus niger, Fusarium venenatum)*, and yeasts (*Saccharomyces cerevisiae, Yarrowia lipolytica*). Several industries and companies have already transformed agro-industrial waste materials into valuable products using microbial fermentation.

Waste materials, such as wheat straw, sugarcane bagasse, fruit peels, and spent grains, can serve as nutrient sources for microbial growth [61]. For example, *Aspergillus oryzae* can ferment food waste into protein-rich biomass. Quorn foods use *Fusarium venenatum* grown on glucose derived from starch waste to produce mycoprotein, a meat alternative [36]. Unilever and Danone have invested in startups exploring microbial protein production from food processing byproducts like potato peels and molasses.

#### 6.3.2. Industrial Byproducts

Waste streams from bioethanol production, dairy processing, and brewery industries contain valuable nutrients that support microbial growth [62]. For example, *Yarrowia lipolytica* can utilize glycerol (a byproduct of biodiesel production) to produce SCP.

#### 6.3.3. Methanotrophic and Photosynthetic Pathways

Bacteria like *Methylococcus capsulatus* can convert methane into microbial protein, reducing greenhouse gas emissions [63]. Microalgae, such as Chlorella vulgaris, use CO_2_ for protein biosynthesis, contributing to carbon sequestration. The microbial fermentation of agricultural and food waste offers a sustainable and economically viable solution for protein production. Table 6 compares different agricultural and food waste substrates, including the types of microorganisms used, conversion rates, protein content, and the economic potential of each substrate for producing microbial protein.

## 7. Scaling Up Microbial Protein Production

**Figure 3:**
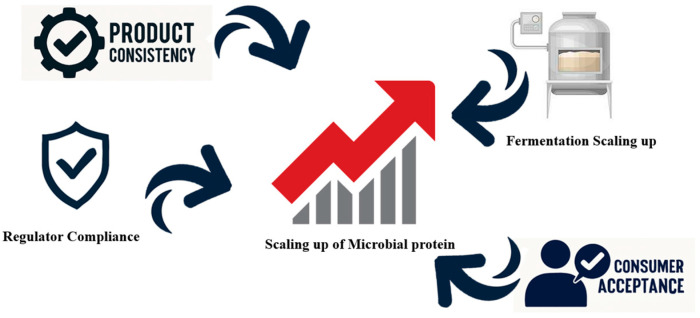
Challenges in scaling up microbial protein production and the solutions.

**Figure 4:**
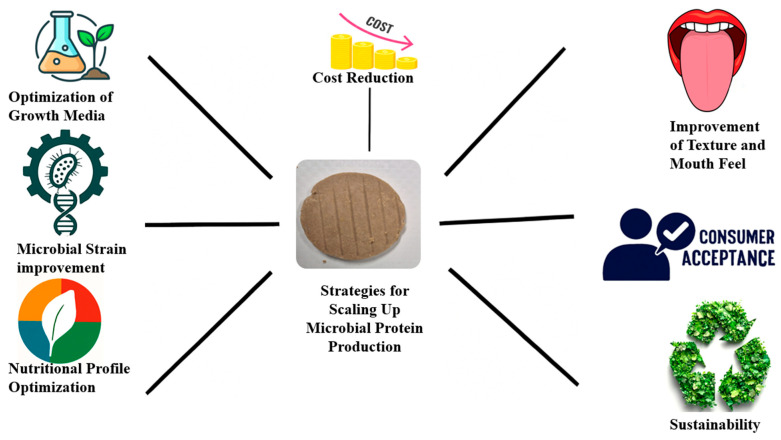
Strategies for scaling up microbial protein production.

For microbial proteins to compete with conventional proteins, large-scale production and cost effectiveness must be achieved [64]. Figure 3 presents the challenges associated with scaling up microbial protein production, along with potential solutions. Furthermore, there are several strategies to improve the scaling up of microbial protein production, which are presented in Figure 4. Several companies are leading in terms of large-scale microbial protein production, as shown in Table 7. Scaling up microbial protein production involves transitioning from laboratory-scale processes to industrial-scale operations, while maintaining product consistency, regulatory compliance, and consumer acceptance, as illustrated in the diagram. The main methods include batch fermentation, fed-batch fermentation, and continuous fermentation. Batch fermentation is simple and easy to control, making it ideal for early-stage production; however, it has lower productivity and longer downtimes. Fed-batch fermentation enables better control over the nutrient supply, leading to higher cell densities and protein yields, but it requires more complex monitoring and can be costly to scale. Continuous fermentation offers the highest productivity and efficient use of equipment, making it suitable for large-scale operations, but it poses challenges in regard to maintaining long-term sterility and consistent product quality. Other innovations like precision fermentation (e.g., using genetically engineered microbes) and solid-state fermentation (using agro-waste as a substrate) are gaining attention for their sustainability and substrate flexibility benefits, although they may face regulatory hurdles or technical limitations in terms of process control.

## 8. Sustainability and Environmental Benefits of Microbial Proteins

Producing microbial proteins presents a sustainable alternative to conventional animal agriculture, offering significant environmental advantages [15]. Compared to livestock farming, microbial protein production requires less land and water, generates lower greenhouse gas (GHG) emissions, and contributes to a circular bioeconomy by utilizing waste streams as feedstocks [65].

One of the most significant advantages of microbial protein production is its minimal land and water footprint compared to conventional meat production [15]. Microbial proteins require 10–100 times less land than traditional livestock farming [15]. Unlike animal agriculture, which depends on extensive pastureland and feed crop cultivation, microbial proteins can be produced in bioreactors, significantly reducing deforestation and habitat loss [65]. Table 8 presents a comparison of land use per kilogram of protein across various protein sources.

### 8.1. Water Conservation

The water footprint of microbial proteins is significantly lower than beef and poultry. Microbial fermentation is conducted in closed systems, minimizing water loss and contamination. Table 9 compares the water conversion efficiency per kilogram of protein among various protein sources.

### 8.2. Lower Greenhouse Gas Emissions Compared to Conventional Meat

Livestock farming is a major contributor to greenhouse gas (GHG) emissions, particularly methane (CH_4_) from ruminant digestion and carbon dioxide (CO_2_) from feed production and land use changes [70]. In contrast, microbial protein production generates significantly lower emissions. Microbial protein production can reduce GHG emissions compared to beef production [15]. Fermentation-based protein production emits less than 1 kg of CO_2_ equivalent per kg of protein, whereas beef production emits up to 33.30 kg of CO_2_ equivalent per kg of protein [71]. Unlike ruminant livestock, mycoprotein (*Fusarium venenatum*) and bacterial SCP (*Methylococcus capsulatus*) produce minimal methane emissions. Specific microbial fermentation systems can utilize CO_2_ as a feedstock, directly converting waste gases into protein [72].

## 9. Role in Circular Bioeconomy (Using Agricultural/Industrial Waste as Feedstock)

Microbial protein production aligns with circular bioeconomy principles by utilizing waste streams and renewable substrates instead of conventional agricultural inputs [19]

### 9.1. Use of Agro-Industrial Waste

Many microbial strains can grow on low-cost, non-food feedstocks, such as agricultural residues (e.g., wheat straw, corn husks, sugarcane bagasse), food waste (e.g., fruit peels, spent grains), and industrial byproducts (e.g., glycerol from biodiesel production, whey from dairy processing). For example, *Yarrowia lipolytica* yeast can efficiently convert waste glycerol into SCP, reducing industrial waste.

### 9.2. Methane and Hydrogen-Based Fermentation

Methanotrophic bacteria (*Methylococcus capsulatus*) can grow on methane from biogas plants or natural gas, reducing GHG emissions, while producing protein [62]. Hydrogen-oxidizing bacteria can convert H_2_ and CO_2_ into biomass, closing the carbon loop.

### 9.3. Minimal Waste and Byproduct Utilization

The closed-loop production of microbial proteins generates minimal waste, and leftover biomass can be repurposed for animal feed, biofertilizers, or bioplastics. The water used in fermentation can often be recycled, reducing resource consumption [73].

Furthermore, the microbial conversion of non-food raw materials into single-cell protein (SCP) represents a transformative approach to sustainable nutrition and waste valorization. Using substrates, such as methane, molasses, potato peels, and citrus pulp, microbial strains like *Rhodococcus opacus, Candida utilis*, and *Methylophilus methylotrophus* can produce protein-rich biomass, with yields far exceeding traditional agriculture. These processes involve substrate pretreatment, controlled fermentation, and post-harvest refinement to reduce the RNA content and enhance digestibility. According to Zhuang et al. [74], SCP production from renewable feedstocks offers high energy efficiency, minimal environmental impacts, and scalability through the use of metabolic engineering and bioreactor optimization. Countries like China, the U.S., Finland, and Brazil are leading in regard to SCP innovation, leveraging agro-industrial waste to create high-value protein for food and feed applications. This technology not only addresses global protein shortages, but also contributes to circular bioeconomy models by transforming waste into nutritional assets. In terms of the global market, a joint report by Boston Consulting Group and Blue Horizon estimates that alternative proteins (including SCP) could account for 22% of global protein consumption by 2035, creating a market worth 300 billion USD [74] and there will definitely be the opportunity to use waste or byproducts in the manufacturing of microbial protein-based meat.

## 10. Regulatory Challenges and Consumer Acceptance of Microbial Proteins

The commercialization of microbial proteins faces several challenges related to regulatory approvals, safety concerns, and consumer acceptance [75]. As microbial proteins gain popularity as sustainable alternatives to conventional meat, policymakers, scientists, and food manufacturers must navigate complex regulations, conduct rigorous safety assessments, and address consumer perceptions to ensure widespread adoption.

### 10.1. Current Regulations for Microbial Protein Commercialization

The regulatory landscape for microbial proteins varies across different regions, requiring extensive safety evaluations and approvals before they can be marketed for human consumption.

#### 10.1.1. United States (FDA Regulations)

The U.S. Food and Drug Administration (FDA) regulates microbial proteins under the Generally Recognized as Safe (GRAS) framework. Companies must provide toxicological, allergenicity, and compositional data to demonstrate the safety of microbial proteins. For example, Quorn™ (mycoprotein from *Fusarium venenatum*) received FDA GRAS approval in 2002 [76].

#### 10.1.2. European Union (EFSA Regulations)

The European Food Safety Authority (EFSA) classifies microbial proteins under the Novel Food Regulation (EU 2015/2283), which includes the following requirements:

🗸 Detailed safety assessments;
🗸 Nutritional evaluations;
🗸 Toxicology and allergenicity tests;
🗸 For example: Solein™ (CO_2_-based microbial protein made by Solar Foods) has received EFSA novel food approval [77].

#### 10.1.3. Other Global Regulations

The United Kingdom: Follows the EFSA guidelines, but has an independent regulatory process post-Brexit. China and India: The regulatory frameworks are evolving, with microbial proteins expected to be reviewed under food safety laws for novel foods. Singapore: A leader in alternative protein approvals, with a streamlined process for novel proteins, including microbial fermentation-based foods.

### 10.2. Safety Concerns and Risk Assessment

Regulatory agencies assess microbial proteins based on their safety profile, potential allergenicity, and processing methods to ensure they meet food safety standards.

#### 10.2.1. Allergenicity and Digestibility Concerns

Fungal mycoproteins (e.g., Quorn™) have been associated with rare allergic reactions in individuals sensitive to molds [78]. The high nucleic acid content in bacterial SCP can increase uric acid levels, posing a risk of gout and kidney stones [42]. Certain processing methods, such as heat treatment and enzymatic digestion, can help reduce nucleic acid levels.

#### 10.2.2. Toxin and Contaminant Risks

Some microbial proteins, particularly those derived from wild-type fungi and bacteria, may produce mycotoxins or endotoxins that must be removed through purification steps [78]. Fermentation residues and residual metabolites must be monitored to ensure that they do not pose health risks.

#### 10.2.3. Genetic Modification and Synthetic Biology

Some microbial proteins are produced using genetically modified (GM) strains to enhance the protein yield and nutritional properties. Regulatory agencies require detailed assessments of GM microorganisms to ensure that they do not introduce harmful genetic elements into the food chain [79].

## 11. Consumer Perception and Market Acceptance

**Figure 5:**
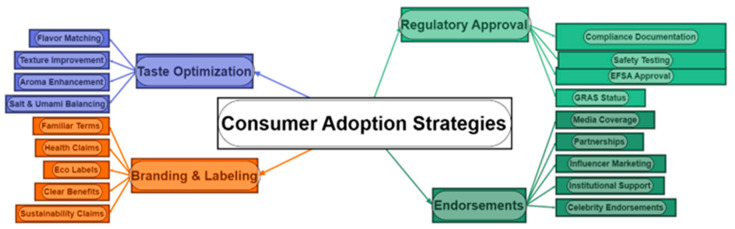
Consumer adoption strategies for improving the market scenario for microbial proteins.

Despite microbial proteins’ environmental and nutritional benefits, consumer acceptance remains a key challenge. Understanding consumer attitudes toward novel foods, safety concerns, and ethical considerations is crucial for market success. Furthermore, key factors that influence the acceptance rate by consumers and strategies to improve the acceptance of microbial proteins are presented in Table 10. Moreover, consumer adoption strategies for strengthening the market scenario in regard to microbial proteins are presented in Figure 5.

### Case Studies of Consumer Response to Microbial Proteins

Quorn™ (mycoprotein from *Fusarium venenatum*) initially faced skepticism from consumers, but gained in popularity due to taste improvements and sustainability marketing [80]. Solein™ (CO_2_-based protein made by Solar Foods) is an eco-friendly protein appealing to environmentally conscious consumers [36]. Calysta’s FeedKind™ products (methane-fed microbial protein for animal feed), which are used as a sustainable fishmeal alternative, are gaining acceptance in the aquaculture industry [81].

## 12. Future Perspectives

**Figure 6:**
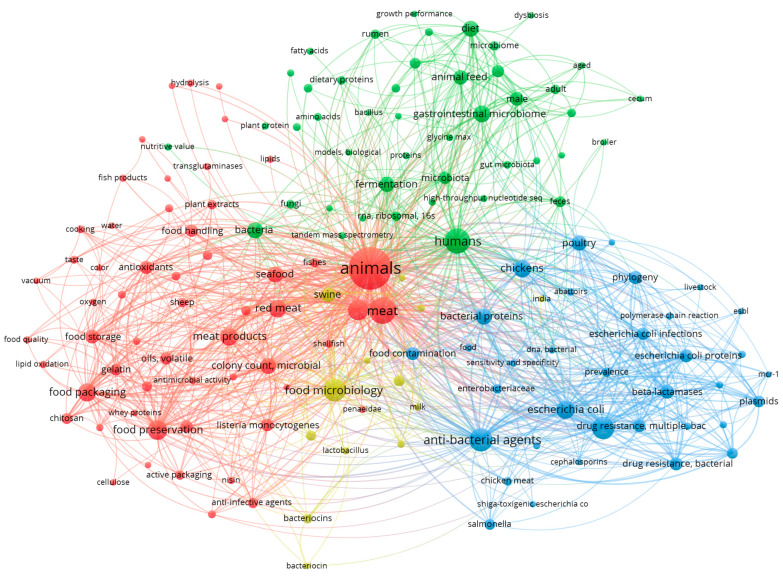
A keyword co-occurrence network, which is generated from the literature published over the past ten years on microbial protein-based meat (derived using the keyword microbial protein in PubMed in the literature published in the last 10 years).

There are several studies that have been conducted on microbial protein-based meat over the last 10 years, as shown in Figure 6. As global food demand increases, microbial proteins have emerged as a sustainable and scalable alternative to conventional protein sources [13]. However, their widespread adoption depends on advances in synthetic biology, precision fermentation, regulatory policies, and investment in large-scale production. Synthetic biology and genetic engineering are transforming microbial protein production by enhancing the protein yield, and enabling functional customization [82]. 

Moreover, genetic modifications have the potential to enhance biomass growth rates, thereby increasing protein production efficiency. Through metabolic engineering, microbes can be optimized to utilize low-cost feed stocks such as CO_2_, methane, or agricultural waste, which can significantly reduce production costs [83]. Additionally, genetic engineering can improve the essential amino acid profiles of microbial proteins, making them more comparable in nutritional quality to animal-derived proteins.

Precision bioengineering allows for the customization of protein structures to improve its texture, digestibility, and taste [84]. Precision fermentation is a revolutionary technology that enables the targeted production of specific proteins, peptides, and functional compounds using microbial systems [85]. Unlike traditional fermentation, precision fermentation inserts specific genes into microorganisms to produce targeted proteins identical to those found in animal or plant sources. Perfect Day uses precision fermentation to produce animal-free dairy proteins (whey and casein) from engineered microbes.

The production of meat-like textures is accomplished by engineering fungal and bacterial proteins to develop enhanced fibrous structures. The creation of hybrid proteins involves combining microbial proteins with plant-based ingredients to improve both taste and functional properties. Additionally, research is focused on generating bioactive peptides from these proteins, which may offer potential health benefits such as anti-inflammatory and immune-modulating effects.

## 13. Conclusions

This review underscores the significance of microbial proteins as a sustainable alternative to meet the increasing global demand for meat, emphasizing their potential to substantially reduce the environmental impact associated with conventional animal-based meat production.

Microbial protein-based meat offers high nutritional value and can be produced with little space compared to animal-based meat production. These advantages make microbial proteins an essential component of future food systems. However, key challenges, such as limited public awareness, high production costs, and regulatory complexities, must be addressed for microbial proteins to achieve large-scale integration. Technological innovations, supportive policy frameworks, and efforts to improve consumer perceptions are crucial to overcoming these limitations. Additionally, restructuring technologies and hybrid formulations that blend microbial and plant-based proteins could help reduce costs and enhance the product’s taste and texture, making microbial protein-based meat analogs more acceptable and economically feasible. Ultimately, microbial proteins represent more than a protein source, they embody a strategic approach to achieving multiple United Nations Sustainable Development Goals, including food security, climate action, and sustainable industry. With continued advancement and collaboration across sectors, microbial proteins could play a transformative role in building a resilient and equitable global food system.

## Figures and Tables

**Table 1:** Connection of microbial protein-based meat with different SDGs.

SDGs	Goal Title	Contribution of Microbial Proteins	Data/Reference
SDG 2	Zero Hunger	Provides high-quality, affordable, and scalable protein to combat food insecurity and malnutrition.	Microbial proteins can be produced using waste substrates like food scraps, reducing the cost of production. A study shows that microbial proteins can provide up to 60% of daily protein requirements at a fraction of the cost of meat-based protein [18].
SDG 9	Industry, Innovation, and Infrastructure	Promotes biotechnology and green industrial growth through sustainable fermentation processes.	Industrial-scale microbial protein production, such as Quorn™ (produced by *Fusarium venenatum*), supports green industry by utilizing sustainable fermentation methods with minimal land use [19].
SDG 12	Responsible Consumption and Production	Enables efficient resource use, minimizes food waste, and supports circular bio-economy practices.	Microbial protein production requires 10–100 times less land and 90% less water than beef [20].
SDG 13	Climate Action	Lowers greenhouse gas emissions by reducing dependence on conventional animal agriculture.	The production of microbial proteins emits less CO_2_ per kg of protein, compared to beef [21].
SDG 15	Life on Land	Reduces land degradation and deforestation by limiting the need for grazing and feed crop cultivation.	Microbial proteins can be produced in bioreactors, requiring minimal land compared to traditional animal farming, which leads to reduced deforestation and soil degradation [22].

**Table 2:** Comparison of microbial protein sources.

Source	Protein Content	Amino Acid Profile	Key Advantages	Example Species	References
Bacteria	50–80%	Complete proteins, high in lysine, threonine, and glutamine	Fast growth, utilizes diverse feedstocks	*Methylophilus methylotrophus*, *Bacillus subtilis*	[26]
Fungi	40–60%	Complete proteins, high in lysine, threonine, glutamic acid	Meat-like texture, rich in fiber (beta-glucans)	*Fusarium venenatum* (Quorn), *Aspergillus oryzae*	[29]
Yeasts	45–65%	Complete proteins, high in B-vitamins, methionine, glutamic acid	High in vitamins, easy to cultivate	*Saccharomyces cerevisiae*, *Candida utilis*	[28]
Algae	50–70%	Complete proteins, high in glutamic acid, lysine, valine, and leucine	Contains essential fatty acids (omega-3), antioxidants	*Spirulina*, *Chlorella vulgaris*	[27]

**Table 3:** Nutritional profile of microbial protein-based meat.

Aspect	Details	Microbial Source(s)	Reference
Protein Content	40–80% of dry weight, depending on species and conditions	Yeast, fungi, bacteria, algae	[26]
Essential Amino Acids	Complete proteins contain all nine essential amino acids	Yeast, mycoprotein, algae, bacteria	[41]
High Lysine and Threonine	Enhances the nutritional value of plant-based diets	Fungal (mycoprotein), bacterial proteins	[42]
Leucine and Valine	Crucial for muscle synthesis and recovery	Algal (*Spirulina, Chlorella*)	[43]
Glutamic Acid	Umami flavor enhances taste in food applications	Yeast (*S. cerevisiae*)	[44]
Digestibility Score	85–90% for mycoprotein; >90% for yeast/bacteria	Quorn™, yeast, bacteria	[29]
Digestibility Challenges	Cell-wall composition (e.g., chitin, polysaccharides) may limit absorption	Algae, fungi	[15]
Improved Digestibility	Processing, like heat treatment and enzymatic hydrolysis	All microbial proteins	[29]
Nucleic Acids	High content may raise the level of uric acid; it is reduced by processing	Bacterial, yeast proteins	[45,46]
B-complex Vitamins	B1, B2, B3, B6, B12, folate	Yeast, algae	[47]
Iron and Zinc	Bioavailable forms support immune and metabolic function	Mycoproteins (*F. venenatum*)	[48]
Omega-3 Fatty Acids	Contains EPA and DHA for cardiovascular health	Algae (*Spirulina, Chlorella*)	[27]
Dietary Fiber and Prebiotics	Chitin, beta-glucans, and insoluble fiber support gut health	Mycoprotein, algae, fungi	[27]
Antioxidants (Phycocyanin)	Reduces oxidative stress, anti-inflammatory	Algae (*Spirulina*)	[27]
Immunomodulatory Peptides	Bioactive peptides with potential antihypertensive effects	Various microbial proteins	[49]
Antioxidants (Phycocyanin)	Reduces oxidative stress, anti-inflammatory	Algae (*Spirulina)*	[27]

**Table 4:** Microorganisms commonly used for SCP production.

Microorganism	SCP Application	Example Products	References
*Fusarium venenatum*	Mycoprotein	Quorn™	[48]
*Methylophilus methylotrophus*	Bacterial SCP	Animal feed	[58]
*Saccharomyces cerevisiae*	Yeast SCP	Nutritional supplements	[20]
*Spirulina platensis*	Algal SCP	Food supplements, protein powders	[59]

**Table 5:** A comparison of different fermentation methods used to produce microbial protein-based meat analogs.

Method	Advantages	Disadvantages	Popularity	Ref.
Submerged Fermentation (SmF)	High yields, scalable, precise control over conditions	High energy costs, expensive substrates, waste generation	Most common technique, especially for yeast and mycoprotein	[52]
Solid-State Fermentation (SSF)	Low energy use, uses agricultural waste, nutrient-rich products	Lower yields, longer fermentation time, process complexity	Although less widely adopted, its use is steadily increasing in the production of sustainable and fungal proteins.	[54]
Gas Fermentation	Sustainable (recycles industrial gases like methane), no need for agricultural land, high protein yield	Requires specialized infrastructure, high capital investment, limited organisms	Gaining increasing attention for its potential in carbon capture, though it remains in the early stages of development	[54]
Photoautotrophic Cultivation	Sustainable, high yield in small areas, uses CO_2_ and wastewater	High cost for infrastructure, limited species for protein production, harvesting can be costly	Growing use, especially for algae-based proteins and health supplements	[51]

**Table 6:** Comparison of agricultural and food waste used in microbial fermentation.

Waste Type	Microorganisms Used	Conversions	Economic Value	Examples
Agricultural Waste	*Aspergillus niger, Trichoderma reesei, Fusarium venenatum*	Conversion of carbon into microbial protein	Waste reduction, low-cost protein source, potential to develop biofuels	Corn stover, wheat straw, sugarcane bagasse
Food Waste	*Saccharomyces cerevisiae, Candida utilis*	Conversion of organic matter into protein	Reduces food waste, cost-effective protein production	Fruit peels (banana, citrus), spent grains (brewers)
Spent Brewing Grains	*Saccharomyces cerevisiae, Aspergillus oryzae*	Microbial protein	Useful for animal feed and human food, utilizes waste	Brewer’s spent grain
Fruit and Vegetable Waste	*Saccharomyces cerevisiae, Aspergillus niger*	Conversion of sugars into protein	Reduces food waste, potential to develop bio-based products	Banana peels, apple cores, potato skins
Oilseed Cakes (e.g., Soy, Sunflower)	*Rhizopus oryzae, Mucor circinelloides*	Conversion of carbon in oilseed cakes into microbial protein	Byproduct utilization, sustainable protein source	Soybean meal, cottonseed residues

**Table 7:** Companies leading large-scale microbial protein production.

Company	Technology	Product
Quorn	Mycoprotein fermentation	Quorn™
Calysta	Methane-based fermentation	FeedKind™ (animal feed)
Solar Foods	CO_2_-based fermentation	Solein™
Nature’s Fynd	Fungal fermentation	Fy™ protein

**Table 8:** Comparison of land use (m^2^ per 100 g of protein) (adopted from the following references).

Protein Source	Land Use (m^2^/100 g Protein)	References
Beef	163.6	[66]
Chicken	7.1	[66]
Soy protein	3.4	[66]
Microbial proteins (e.g., mycoprotein)	<1	[67]

**Table 9:** Comparison of water use (liter per kg of protein) (adopted from the following references).

Protein Source	Water Use (L/kg protein)	References
Beef	8849	[68]
Pork	4050	[68]
Poultry	2337	[68]
Microbial proteins	0.5–1.45	[69]

**Table 10:** Key factors influencing consumer acceptance and strategies to improve the acceptance of microbial proteins.

Factor	Consumer Concern	Strategies to Improve Acceptance
Familiarity and Awareness	Lack of knowledge about microbial proteins	Public education campaigns, clear labeling
Taste and Texture	Concerns about texture or flavor differences compared to meat	Flavor enhancement, improved processing
Safety and Naturalness	Concerns about “unnatural” or lab-grown foods	Transparency in terms of the production process, safety certifications
Sustainability Benefits	Consumers may not prioritize sustainability	Clear communication of environmental benefits
Price and Availability	Higher cost than conventional proteins	Economies of scale, government incentives
